# Gut Microbiome, Functional Food, Atherosclerosis, and Vascular Calcifications—Is There a Missing Link?

**DOI:** 10.3390/microorganisms9091913

**Published:** 2021-09-09

**Authors:** Dragos Cretoiu, Ruxandra Florentina Ionescu, Robert Mihai Enache, Sanda Maria Cretoiu, Silviu Cristian Voinea

**Affiliations:** 1 Department of Morphological Sciences, Cell and Molecular Biology and Histology, Carol Davila University of Medicine and Pharmacy, 8 Eroii Sanitari Blvd, 050474 Bucharest, Romania; dragos@cretoiu.ro; 2 Alessandrescu-Rusescu National Institute for Mother and Child Health, Fetal Medicine Excellence Research Center, 020395 Bucharest, Romania; 3 Central Military Emergency Hospital “Dr. Carol Davila”, Department of Cardiology I, 134 Plevnei Blvd, 010825 Bucharest, Romania; uxandra-florentina.ionescu@rez.umfcd.ro; 4 Department of Radiology and Medical Imaging, Fundeni Clinical Institute, 258 Fundeni Road, 022328 Bucharest, Romania; robert-mihai.enache@rez.umfcd.ro; 5 Department of Surgical Oncology, Prof. Dr. Alexandru Trestioreanu Oncology Institute, Carol Davila University of Medicine and Pharmacy, 252 Fundeni Road, 022328 Bucharest, Romania; dr.voinea_silviu@yahoo.com

**Keywords:** atherosclerosis, vascular calcification, functional food, microbiome, gut microbiota

## Abstract

The gut microbiome is represented by the genome of all microorganisms (symbiotic, potential pathogens, or pathogens) residing in the intestine. These ecological communities are involved in almost all metabolic diseases and cardiovascular diseases are not excluded. Atherosclerosis, with a continuously increasing incidence in recent years, is the leading cause of coronary heart disease and stroke by plaque rupture and intraplaque hemorrhage. Vascular calcification, a process very much alike with osteogenesis, is considered to be a marker of advanced atherosclerosis. New evidence, suggesting the role of dietary intake influence on the diversity of the gut microbiome in the development of vascular calcifications, is highly debated. Gut microbiota can metabolize choline, phosphatidylcholine, and L-carnitine and produce vasculotoxic metabolites, such as trimethylamine-N-oxide (TMAO), a proatherogenic metabolite. This review article aims to discuss the latest research about how probiotics and the correction of diet is impacting the gut microbiota and its metabolites in the atherosclerotic process and vascular calcification. Further studies could create the premises for interventions in the microbiome as future primary tools in the prevention of atherosclerotic plaque and vascular calcifications.

## 1. Introduction

The human body is populated by a multitude of microorganisms, ranging from bacteria and viruses to fungi. The most abundant and complex community of microbes is found in the gastrointestinal system, known as the gut microbiome [1]. There are five phyla that dominate the gut microbiome: *Firmicutes*, *Bacteroidetes*, *Actinobacteria*, *Proteobacteria*, and *Verrucomicrobia* [2,3]. Physiological functions of the gut microbiome are fermentation [4], vitamin synthesis [5], energy production through short-chain fatty acids (SCFAs) [6], and adjustment of the intestinal mucosal barrier [2,7]. SCFAs are directly responsible for gut–brain signaling, while bile acids (BA) and trimethylamine-N-oxide (TMAO) are associated with the atherosclerotic process [2,8,9]. 

**Figure 1:**
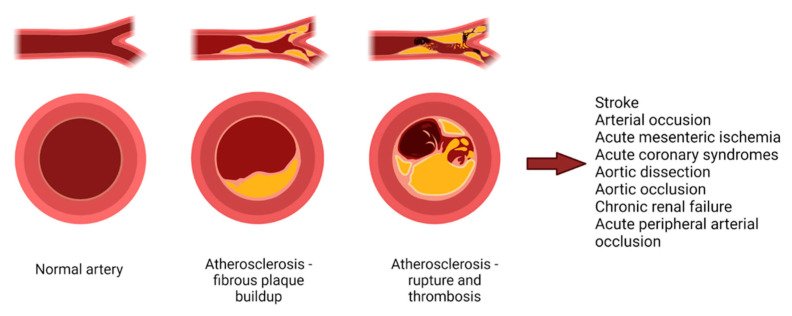
Evolution of atherosclerosis and its consequences. The process of atherosclerosis begins with an endothelial injury, leading to accumulation of macrophage foam cells and infiltration of smooth muscle cells with development of fatty streaks. From this point, atherosclerosis, through an inflammatory process, will lead to the development of a vulnerable plaque, consisting of necrotic core, micro- and macrocalcification, and intraplaque hemorrhage. When the thin fibrous cap of the vulnerable plaque develops a fissure, platelets will begin to aggregate, leading to thrombosis and acute coronary syndrome, but also other pathologies. Created with BioRender.com (Last accessed on 21 July 2021).

Atherosclerosis, a disease affecting the arteries, is the leading cause of death by heart disease and stroke (~50% of all deaths in Western societies), while cardiovascular diseases (CVDs) represent 32% of all global deaths in 2019 [10]. Atherosclerosis can be regarded not only as an unavoidable degenerative result of aging, but also as a chronic inflammatory disorder, with possible acute complications, such as plaque rupture and thrombosis [11]. Both genetic and environmental factors can influence this condition. Atherosclerosis (and intimal calcifications) and arteriosclerosis (and medial calcifications) are intertwined, precipitating vascular impairment [12]. The evolution of atherosclerosis and its consequences are illustrated in Figure 1. The initial lesions of atherosclerosis are represented by subendothelial accumulations of macrophages containing cholesterol (foam cells). Fatty band deposits are the precursors of fibrous lesions, which contain necrotic debris rich in lipids and smooth muscle cells (SMCs). The fibrous cap of the plaque is represented by SMCs and extracellular matrix, which encapsulates a necrotic core. Plaques can evolve through calcification, ulceration, and hemorrhagic processes. Critical outcomes of plaque evolution can consist of acute occlusion of the artery through thrombus or clot formation, leading to stroke or myocardial infarction, especially if located in the coronary arteries [13]. Thrombosis of the plaque can be linked to erosion or breach of the lesion [11]. Animal studies revealed that deficiency of apolipoprotein E (apoE) or low-density lipoprotein (LDL) receptors can lead to advanced atherosclerotic lesions [14].

“Monckeberg sclerosis” is defined as the calcification of the medial layer of arteries, in the absence of atheroma, being more frequently located in the lower limb arteries, but also in the aorta. The calcification process affecting elastic or muscular arteries is ectopic and is usually observed in patients with chronic inflammatory conditions such as diabetes mellitus, chronic kidney disease (CKD), and hypertension. There can also be a genetic component involved [12]. 

This review analyzes the main findings related to gut microbiota involvement in the development of CVD, with an emphasis on the direct relation with the calcification of the atherosclerotic plaques. The roles of diverse macro- and micronutrients are also discussed, as well as the most recent findings related to the use of different probiotics as future therapies for CVD.

## 2. General Mechanisms of Vascular Calcification

Vascular calcifications occur frequently in the large and medium arteries, affected by atherosclerosis, either in the intima (focal plaques) or in the media. Calcifications can also occur in arteries without atherosclerosis, e.g., calcification of internal elastic lamina in patients infected with HIV [15]. These types of arterial calcifications not only vary morphologically and epidemiologically, but they also have different clinical significance [12]. Features that can favor vascular calcifications can be aging, male sex, hypertension, diabetes mellitus, CKD, dyslipidemia, and smoking. In addition, inflammation, oxidative stress, shear stress, advanced glycation end-products, elevated calcium-phosphate product, high angiotensin II concentrations, uremic toxins, and vitamin K deficiency (or antagonists) have significant effects on the vascular system [16].

Calcium deposits at intimal levels, diffuse or punctiforme, in the presence of atherosclerosis, are frequently found in coronary arteries and aorta. They are associated with the accumulation of lipids and macrophages [12,17]. Lipid accumulation and atherosclerotic plaque development can lead to the calcification of the intima, on the grounds of the inflammatory response. Both intimal and medial layer calcifications lead to increased vascular stiffness, diminishing the capacity to adapt to variations in blood pressure and therefore promoting injury and advancement of atherosclerotic plaques [12]. Considered for a long time to be a passive process, vascular calcification was demonstrated to be an actively regulated phenomenon, which resembles bone development. Several bone-regulating proteins, such as osteopontin, matrix Gla protein, osteocalcin, osteonectin, collagen I and II, alkaline phosphatase, bone sialoprotein, and bone morphogenic proteins are expressed at the level of human vascular smooth muscle cells (VSMCs) [17]. Several substances that can either favor or inhibit vascular calcifications in CKD are depicted in Table 1. Vascular calcifications can be considered predictive indicators for CVDs and are associated with a poor prognosis, including higher risk of mortality by adverse cardiovascular events (stroke, acute myocardial infarction, peripheral vascular diseases).

## 3. Particularities of Gut Microbiota in Atherosclerosis

Three different enterotypes were identified depending on the dominant genera: enterotype 1 dominated by *Bacteroides*, enterotype 2 dominated by *Prevotella*, and enterotype 3 dominated by *Ruminococcus* [26]. *Bacteroides* dominance was associated with the habits of eating animal protein, amino acids, and saturated fats [27]. *Prevotella* was found to be in lower levels in these patients, but in high concentrations in patients with diets rich in carbohydrates and simple sugars [27]. Individuals with diets low in fat and animal protein, but high in fiber, starch, and plant polysaccharides represent another category presenting abundant *Prevotella* [28]. Animal studies pointed out that diets high in unsaturated fats increase *Actinobacteria (Bifidobacterium)*, lactic acid bacteria (*Lactobacillus* and *Streptococcus*), and *Verrucomicrobia (Akkermansia muciniphila)* [29,30], but also amplify the translocation of lipopolysaccharides [31]. Fruits and vegetables, naturally abundant in polyphenols [32], alongside probiotics, can raise levels of *Bifidobacterium* and bacteria that produce lactic acid and diminish the populations of enteropathogenic bacteria [30].

The adult microbiome is attracting more and more interest because there seems to be a link between the degree of dysbiosis and various pathologies, including cardiovascular diseases [33].

Symptomatic atherosclerotic patients seem to display numerous populations of *Collinsella* compared to healthy people [2,34]. Some studies concluded that the gut microbiome of patients with atherosclerosis had an abundance of *Streptococcus* spp. and 4 nterobacteriaceae (including *Escherichia coli*, *Enterobacter aerogenes*, and *Klebsiella* spp.). As a result, metabolism and molecule transport, including TMAO, are affected. Furthermore, the gut microbiome of patients with CVDs is more proinflammatory [2,35]. Studies showed the bacterial phylotypes found in the atherosclerotic plaque were like the ones in the oral cavity, especially in patients with periodontal disease. Moreover, dysbiosis is correlated with plasma cholesterol levels [36].

**Figure 2:**
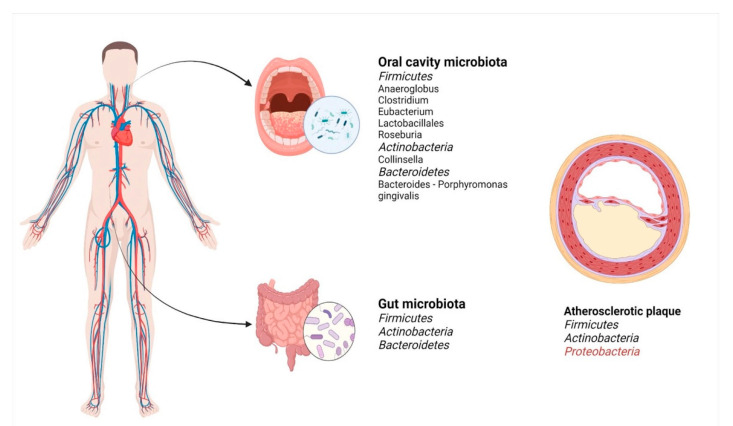
Main bacterial phyla found in atherosclerotic plaque. Bacterial analysis from atherosclerotic plaque samples proved the existence of microorganisms common with the gut microbiota or oral cavity microbiota. The oral cavity and the gut are dominated by *Firmicutes*, while atherosclerotic plaques are rich in *Proteobacteria*. The *Bacteroidetes* phylum is very well represented in the gut but is present to a lesser extent in atherosclerotic plaques. The ratio of *Firmicutes/Bacteroidetes* is demonstrated to be higher in patients suffering from coronary artery disease. Created with BioRender.com (Last accessed on 21 July 2021).

The main phyla and genera present in the oral cavity, gut, and atherosclerotic plaque can be observed in Figure 2. Patients suffering from coronary artery disease have dysbiosis, displaying increased levels of *Lactobacillales* order (*Firmicutes* phylum—*Lactobacillus*, *Streptococcus*, and *Enterococcus* genera) alongside a decrease in *Bacteroidetes* phylum (*Bacteroides* and *Prevotella* genera) [37]. Several studies found in this type of patient low concentrations of *Faecalibacterium* (possessing an anti-inflammatory role [38,39]), *Subdoligranulum*, *Roseburia* (involved in SCFA production [40,41]), *Eubacterium rectale*, and numerous populations of *Escherichia/Shigella* and *Enterococcus* [42]. Elevated bacterial diversity was described in patients with coronary artery disease. *Staphylococcus* species, *Proteus vulgaris*, *Klebsiella pneumoniae*, and *Streptococcus* spp. were identified in atheromatous plaques. Nevertheless, a definitive causal role between the gut microbiota composition and atherosclerosis was not clearly established. It can be affirmed, though, that the presence of certain bacteria may be involved with atherosclerosis evolution [43].

## 4. The Role of Inflammation and Dysbiosis in the Atherosclerotic Process

The human gut microbiota varies throughout life and with dietary changing patterns. Dysbiosis can lead to inflammatory or immune disorders by activating proinflammatory responses throughout the body. Gut dysbiosis is linked to dyslipidemia. Atherosclerosis is represented by a process of chronic inflammation, alongside lipid accumulation in vascular walls. The gut microbiome dysbiosis can accentuate low-grade inflammation at intestinal level, favoring the passage of bacterial and metabolism products into the bloodstream, perpetuating chronic inflammation [44].

There is evidence pointing out that cells of the immune system, from carotid or coronary atherosclerotic plaques in patients with acute coronary syndromes, can generate antibodies against gut microbial antigens, such as *Klebsiella* and *Proteus* [45]. Furthermore, inflammation is indicated to play an important role in the development of vascular calcifications [2,46]. Moreover, the composition of atherosclerotic plaques is like the gut or oral cavity microbial load, as previously discussed here [36,47].

Diet patterns can influence the composition of gut microbiota, which in turn can influence the general health of the individual by digestion and absorption of nutrients [48]. This way, the dietary pattern could represent one of the most uncomplicated and straightforward methods to change the human microbiome [27]. Representative of the Western diet is the high protein and fat intake, elevating the cardiovascular risk and diminishing *Bifidobacterium* populations [49]. In comparison, the Mediterranean diet is abundant in vegetables, grains, fruits, antioxidants, and fibers [50]. This diet can lower oxidative stress and inflammation, and accentuate antioxidant effects and nitric oxide bioavailability, thus ameliorating vascular and cardiac function. It can also be associated with a lower incidence and severity of heart failure [51].

## 5. The Microbiome, Dietary Nutrients, and Vasculotoxic Metabolites

**Figure 3:**
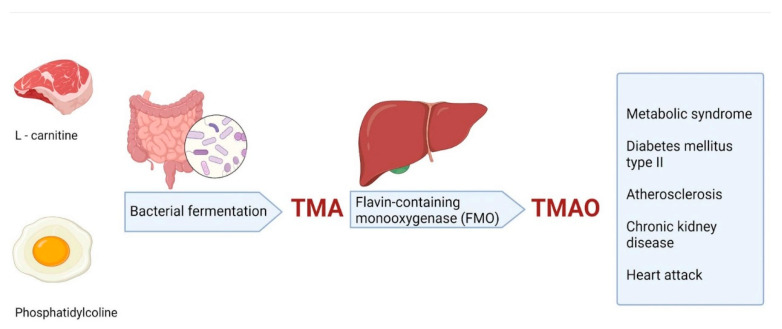
The TMA/FMO/TMAO pathway is highly dependent on the diet constituents and plays a role in the pathogenesis of CVD. Trimethylamine (TMA), resulting from bacterial metabolism, will interact with the enzyme flavin monooxygenase 3 (FMO), which is a powerful modifier of cholesterol metabolism and responsible for trimethylamine N-oxide (TMAO) production. Created with BioRender.com (Last accessed on 21 July 2021).

The link between the intestinal microbiota and atherosclerosis has underlined the importance of dietary changes as a new method of vascular prevention [52]. In the past, cholesterol and saturated fats were at the forefront of nutritional management, but now it is established that lowering the intake of L-carnitine from red meat and phosphatidylcholine from egg yolks can also play a key role in the prevention of CVDs, including atherosclerosis (Figure 3) [52]. If so far, drugs such as statins were used as a first intention in lowering fasting LDL levels, studies have pointed out that after a high-cholesterol meal, arterial inflammation, oxidative stress [53,54], and endothelial dysfunction [55] increase for several hours [56].

The microbiota is strongly connected to our health by modulating, among others, the immune function, intestinal function, and bioactivation of nutrients and vitamins. Recently, studies have shown that intestinal microbiota produce vasculotoxic metabolites from dietary components [57]. The most studied pathway that links vasculotoxic metabolites of the microbiota to CVDs, including atherosclerosis, is the conversion of carnitine (found mainly in red meat) and phosphatidylcholine (from egg yolk) into trimethylamine (TMA) by intestinal microorganisms (Figure 3) [58,59].

Phosphatidylcholine, under the action of intestinal lipases, produces a variety of metabolic products, including the choline-containing nutrients glycerophosphocholine, phosphocholine, and choline. Further, these are metabolized by the gut microbiota, mainly from cecum and large bowel to TMA, which is oxidized by hepatic flavin monooxygenases (FMOs) to trimethylamine N-oxide (TMAO). TMAO, a proatherogenic compound, can stimulate macrophage scavenger receptors, increasing the amount of cholesterol in macrophages, the accretion of foam cells in artery walls, and, therefore, atherosclerosis [59]. Additionally, choline can be oxidized in the liver and kidneys to betaine, which represents another way for bacteria to form TMA [60].

L-carnitine consumption has increased in recent years in developed countries and represents a major risk factor for CVDs. It contains a TMA structure, like that of choline, that can activate the same pathogenic pathway [59].

As an assumption, other dietary components that have a TMA structure can also produce TMAO from gut microbiota and lead to atherosclerosis [60]. Consequently, it is important for future studies to raise awareness about this hypothesis and verify its veracity.

Vasculotoxic metabolites are filtered by the kidney and excreted into urine, so this must be considered in the case of patients with renal failure, as they must limit red meat and egg yolk ingestion. Otherwise, levels of TMAO increase, leading to a severe decline in glomerular filtration and a higher cardiovascular risk [61]. The intestinal microbiota also generates other metabolic products that can increase cardiovascular risk in these patients, such as indoxyl sulfate, indole-3-acetic acid, and p-cresyl sulfate [60,62].

Uremic toxins, represented by indoxyl sulfate [63] and indole 3-acetic acid [60], metabolized from tryptophan ingestion and serum p-cresyl sulfate [64], respectively, are accumulated in case of renal failure. They increase the concentration of reactive oxygen species (ROS) in endothelial cells, contributing to endothelial dysfunction, resulting in aortic calcification, vascular stiffness, and higher cardiovascular mortality [65].

An interesting aspect is that vegans lack the intestinal bacteria that produce trimethylamine (TMA), so the consumption of L-carnitine among vegans does not lead to an increased amount of TMAO [59].

**Figure 4:**
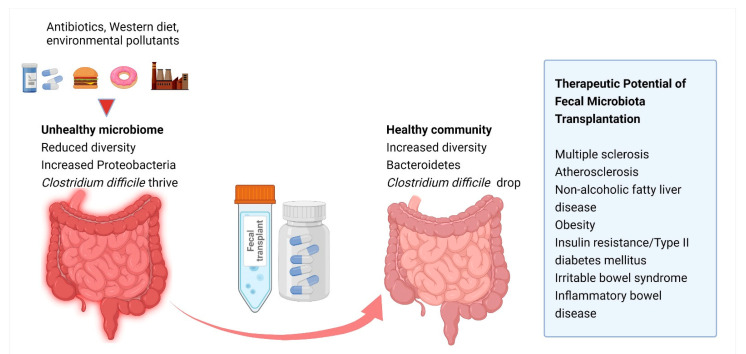
Beneficial effects of fecal microbiota transplantation. The concept of fecal transplantation is represented by the transfer of beneficial bacteria from the stools of a healthy donor to a patient with a disease caused by dysbiosis of their gut microbiota, in order to increase microbial diversity. Created with BioRender.com (Last accessed on 21 July 2021).

Studies have underlined the hypothesis that the intestinal microbiota is adjustable [66] and one can speculate that stool transplantation, already used in the treatment of *Clostridium difficile* infections, could be useful in the prevention of atherosclerosis. However, evidence is conflicting regarding the subject. For instance, the study of Smits et al. pointed out that levels of TMAO and vascular inflammation markers are not influenced by fecal microbiota transplantation in patients with metabolic syndrome [67]. The concept of fecal transplantation and its other possible beneficial effects are illustrated in Figure 4.

## 6. The Contribution of Dietary Factors to Vascular Calcification

Cardiovascular calcifications are a predictor of cardiovascular events and mortality. Since there is no accurate treatment for cardiovascular calcifications, studies regarding the relationship between diet and the vascular calcification process were also considered over time. Some of the results are summarized in Table 2.

Establishing connections between the effects of certain foods and the development of atherosclerosis is a difficult process, due to the impossibility of forming batches to be subjected to the same living conditions and appropriate controls. The atherosclerotic process is influenced by numerous factors, including circulating lipid levels and the co-existence of chronic inflammation. Among other interventions in the management of atherosclerosis one can also include the functional foods, a term coined in Japan in the early 1980s. Functional foods are generally defined as edibles with physiological advantages, and which are responsible for short- or long-term benefits, other than their nutritional values [94]. Moreover, other terms were introduced, such as nutraceuticals or bioceuticals, known as a food or part of a food, that can sometimes be seen as dietary supplements providing health benefits (prevention and even treatment of a specific disease) [95,96]. In CVDs, the need for some nutrients may increase. Several functional foods were demonstrated to have beneficial effects in CVDs by lowering the total lipid concentration (and LDL cholesterol), through their antioxidative properties and by their effect on homocysteine levels (for extensive reviews see [97,98]).

Tomatoes, very rich in lycopene with antioxidant properties, are considered very useful in the maintenance of endothelial function and normal levels of blood glucose and lipids [99]. Cranberries, rich in polyphenols, isoprenoids, and other components, were shown to contribute to an increased resistance of LDL cholesterol to oxidation, inhibit platelet aggregation, and reduce blood pressure [100,101]. Relevant findings indicate the benefits of a diet rich in oily fish, long-chain omega 3 fats, and vegetables, while the avoidance of trans fats and simple sugars might also be protective [68].

Soluble fibers such as pectins from apples, pears, and potatoes are important because they can bind to cholesterol in the gastrointestinal tract and increase lipid excretion in feces, while β-glucans, found in oats and barley, are associated with a lower incidence of dyslipidemia, hypertension, and obesity [102,103]. Moreover, fibers from flaxseed and psyllium were proved to lower LDL cholesterol [104].

Polyphenols are nutrients that can be found in tea, coffee, wine, fruits, vegetables, cocoa, and mushrooms [105]. They can inhibit oxidases, decrease the production of superoxide and oxidized low-density lipoprotein, suppress VSMCs proliferation and migration, and diminish platelet aggregation, this way lowering the production of reactive oxygen species [105].

Polyphenols can be listed as flavonoids (the most abundant category), phenolic acids, stilbenes, and lignans [105,106]. They can restrain the evolution of arterial hypertension, diabetes mellitus, hyperlipidemia, and obesity [105].

Data from the literature suggest that polyphenols possess anti-inflammatory and antioxidant properties, being able to attenuate the atherosclerotic process in certain conditions [107]. The study of Loke and colleagues indicated that quercetin and theaflavin may reduce the formation of atherosclerotic lesions in Apo E -/- mice [107]. Other studies reveal the property of polyphenol-rich beverages to attenuate the atherosclerotic process in Apo E -/- mice, such as red wine, dealcoholized red wine [108,109], and tea [107,110].

Other micronutrients can display protective roles against cardiovascular calcifications: magnesium (≥380 mg/day) [68,75,76,77,78], phylloquinone and menaquinone (500 μg/day) [68,79,80,82,83], and 25(OH) vitamin D ≥ 75 nmol/L [68]. On the contrary, inorganic phosphorus from food preservatives may trigger the process of calcification [84,85,86]. In CKD patients, VSMCs transdifferentiation can be amplified by hyperphosphatemia, leading to the formation of an osteoid matrix containing calcium at the level of vascular media [111]. A plasmatic homocysteine level of >12 µmol/L could predict cardiovascular calcifications, with accelerated atherosclerotic plaque progression [70]. Even though oxidative stress can initiate vascular calcifications, antioxidant vitamins did not prove their efficacy, α-tocopherol even being able to elevate the risk for calcification [87]. However, other antioxidant substances, such as quercetin [90], resveratrol (red wine) [91], and epigallocatechin gallate (green tea) [68], were protective against the calcification process [68].

A cross-sectional study conducted by Machado et al. indicated a positive association between the dietary intake of calcium, phosphorus, and potassium and coronary artery calcification in patients suffering from CKD [112].

The relationship between calcium intake and cardiovascular disease is highly debated [113,114,115]. Evidence from clinical trials suggested that there could be an association between calcium dietary supplementation and a high risk for cardiovascular events, including myocardial infarction [116,117,118,119]. Calcium could influence the pathogenesis of cardiovascular events by acting on several pathways: lipid metabolism, inflammation, insulin sensitivity and secretion, thrombosis, and vascular calcification [119,120]. Moreover, a daily dose of more than 1400 mg of calcium supplement was associated with increased death rates from all causes, including from CVDs [119,121]. According to the study conducted by Anderson et al. over a 10-year follow-up period, calcium dietary supplementation was independently associated with incident coronary artery calcification, regardless of adjustment for total calcium intake. Nevertheless, a protective link between total calcium intake and incident coronary atherosclerosis, especially among non-supplement users, was shown [119]. Even so, calcium supplementation does not necessarily promote vascular calcifications in patients not suffering from renal disease or hyperparathyroidism [68,71,72,73].

Sun and colleagues conducted studies on ApoE-deficient mice and showed that low dietary potassium favored atherosclerotic vascular calcification and elevated aortic stiffness versus normal potassium-fed mice [88]. On the contrary, high dietary potassium lowered vascular calcification and aortic stiffness. How can that be explained? Diminished potassium concentrations, near the lower limit of the normal range, elevated intracellular calcium, thus activating cAMP response element-binding protein (CREB). CREB can accentuate autophagy and favor VSMCs calcification. VSMCs calcifications were reduced through the inhibition of calcium signals and downscaling of CREB or ATG7, an autophagy regulator (autophagy-related 7 protein). At the level of calcified arteries and aorta of low-potassium-diet-fed mice, high autophagy and CREB signaling were noted [88]. Moreover, a population-based study revealed that elevated dietary potassium levels diminished the proliferation of VSMCs, lowered monocyte adherence to vascular walls, and decelerated the evolution of atherosclerosis [88,89].

Concerning magnesium, in patients with advanced CKD was noted an association between low serum magnesium concentrations, frequent vascular calcifications, and elevated cardiovascular mortality. The results indicate that magnesium could protect VSMCs against calcifications through molecular mechanisms. Oral magnesium supplementation could lead to decreased vascular calcifications in patients suffering from CKD [81].

Regarding vitamin administration, supplements with vitamins A, B, C, D, and E do not seem to possess effects against calcification. Vitamin K1, K2, and magnesium supplementation, alongside aged garlic extract, could possibly slow the progression of calcifications [16].

Vitamin K is an essential cofactor for the activation of some extracellular matrix proteins, including matrix Gla-protein (MGP), which can inhibit vascular calcification. The vitamin K “concept” is represented by structurally related elements, which comprise phylloquinone (vitamin K1), one of the most utilized synthetic nutritional supplements, and menaquinones (vitamin K2) [16,83]. Newer vitamin K supplements were introduced, mostly containing menaquinone 4 (MK-4) and menaquinone 7 (MK-7: the most hydrophobic variant, with good bioavailability and long half-life) [16,83].

The role of vitamin K was also underlined by the study of McCabe and colleagues, which involved rats with adenine-induced chronic renal failure. High dietary vitamin K in rats with CKD decreased the evolution of warfarin-induced calcifications [122]. What is more, vitamin K1 can be transformed into menaquinone-4 [123]; as a result, dietary supplementation can be achieved with vitamin K2 or analogues, instead of high doses of vitamin K1 [16].

Vitamin K antagonists (VKA), among the most recommended oral anticoagulants, influence the regeneration of vitamin K1 and K2, needed for the activation of coagulation factors and matrix Gla protein, which in turn is responsible for the inhibition of arterial calcification. The study conducted by Hasific et al., evaluating the relationship between VKA treatment and the extent of coronary artery calcifications, showed that a longer period of treatment with VKA, but not novel oral anticoagulants, was associated with the risk of a higher category of coronary artery calcification in patients without previous CVDs [124].

Results from the CARDIA study point out that low-carbohydrate diets adopted from young ages are associated with a higher risk of developing coronary artery calcifications, especially when replacements for carbohydrates are animal proteins or fats [69].

Evidence concerning hyperhomocysteinemia, folic acid, and vitamin B12 as indicators for cardiovascular disease and cardiovascular mortality in CKD patients is conflicting. They were suggested to be considered risk factors for the progression of CKD, representing a potential therapeutic target. The pathophysiological chain of events in this condition can result in malnutrition, anorexia, gastroparesis, and diminished intestinal transit, which further reduce serum folic acid and vitamin B12 concentrations [125]. Reduced blood levels of vitamin B12 were associated with elevated body mass index, diabetes mellitus or insulin resistance, dyslipidemia, and CVDs [125,126]. Hyperhomocysteinemia has been noted as a risk factor for CVDs, but the data are still very debated [125,127,128,129]. High homocysteine blood levels, as well as folic acid and vitamin B12 metabolism abnormalities, can be found in patients suffering from CKD [125,130,131]. Results of a meta-analysis summing up to 5123 patients state that hyperhomocysteinemia is a risk factor for cardiovascular disease and mortality for the CKD patients who do not receive folic acid supplementation [125,132]. Hyperhomocysteinemia was also considered a cardiovascular risk factor in several studies [133,134,135,136,137,138,139]. On the other hand, opposite conclusions were drawn by other studies; for example, Suliman et al. [140] and Wrone et al. [141].

Soohoo et al. [142] indicate that low folic acid concentrations are associated with increased all-cause mortality in hemodialysis patients, therefore folic acid supplementation can diminish cardiovascular events, according to Righetti et al. [125,143]. What is to be done regarding diet supplementation? Data from the literature point into different directions concerning this topic. Several studies concluded that folic acid and vitamin B12 supplementation had no impact on all-cause mortality or cardiovascular events in CKD patients [144,145,146], while 5 mg folic acid supplementation in hemodialysis patients reduced cardiovascular events [125,143].

## 7. Pharmacological Approaches Impacting Microbiome, Atherosclerosis, and Vascular Calcifications

Calcium channel blockers and renin–angiotensin–aldosterone inhibitors proved to be able to decrease vascular calcifications in animal models [16,147,148,149,150,151]. Human studies, however, did not provide an accurate conclusion regarding this phenomenon [152,153,154,155]. Statins, although targeting cardiovascular morbidity and mortality, did not modify the evolution of coronary calcifications [156].

Up to now, there is conflicting evidence regarding possible interventions in the gut microbiome to diminish the intensity of atherosclerosis and vascular calcifications.

Patients with CKD represent a population of high interest. In CKD, as the glomerular filtration rate decreases, the colon takes up a bigger role in the excretion of urea and uric acid. Long exposure to high concentrations of urea can lead to excessive development of bacteria containing urease, generating high ammonia levels and increasing the intestinal pH [157]. Elevated concentrations of uric acid and high oxalate secretion in the digestive tract results in an elevated number of uricase-producing bacteria [158,159].

Correction of gut dysbiosis can improve the gut–vascular–bone axis in CKD patients, diminishing vascular calcification development and bone demineralization. Possible solutions can be diet changes, with high fiber intake, probiotics and prebiotics administration, and vitamin K supplementation, as a risk factor for vascular calcification and bone demineralization can be vitamin K deficiency, a common phenomenon in CKD [159,160]. Evidence suggests lower uremic toxin production because of probiotics administration, alongside lower levels of inflammation biomarkers and oxidative stress [161]. Prebiotics (prebiotic-resistant starch supplementation; oat β-glucan) have been linked to the minimization of uremic toxins and inflammatory markers in hemodialysis patients [162,163].

Several clinical studies presented the positive effect of probiotics [164] on blood lipid profile (*Lactobacillus acidophilus*, *Bifidobacterium lactis*, *Lactobacillus plantarum*, *Lactobacillus helveticus*, *Enterococcus faecium*) [165] and underlined their antioxidant effect (*Lactobacillus fermentum*, *Lactobacillus plantarum*, *Lactobacillus acidophilus*, *Lactobacillus casei*, *Lactobacillus reuteri*, *Lactobacillus delbrueckii* ssp. *bulgaricus*, *Lactobacillus sporogenes*, *Lactococcus lactis*, *Lactobacillus bulgaricus*, *Lactobacillus gasser*, *Lactobacillus rhamnosus*, *Lactobacillus langum*, *Lactobacillus paracasei*, *Bifidobacterium lactis*, *Bifidobacterium bifidum*, *Bifidobacterium longum*, *Bifidobacterium BB-12*, *Bifidobacterium infantis*, *Bifidobacterium breve*) [166], but not the amelioration of endothelial function. A promising new treatment for patients at risk could be, according to Matsumoto M., bacterial metabolites control. Some articles also suggested that oral supplementation with polyamines can be efficient in preventing CVDs [167].

Polyamines, spermidine and spermine for example, are bioactive compounds that can enhance autophagy and diminish inflammation, but also possess anti platelet aggregation properties [167,168]. Spermine can inhibit the inflammatory response through restraining proinflammatory cytokine production [167,169]. It can also alter the transmigration of inflammatory cells to the setting of inflammation by suppressing LFA-1, which binds to endothelial cells through intercellular adhesion molecule 1 [167,170].

Spermidine, according to LaRocca et al., elevates the bioavailability of nitric oxide, diminishing oxidative stress, altering structural factors, and promoting autophagy, thus having a powerful anti-aging influence on arteries [167,171]. Autophagy induced by spermidine can prevent atherosclerosis. Another study pointed out that spermidine supplementation, through raising autophagy in aged and salt-sensitive hypertensive animal models, prolonged the lifespan of mice, provided protective effects on the cardiovascular system, lowered blood pressure, and delayed the progression to heart failure [167,172]. Michiels et al. affirmed that spermidine supplements diminished atherosclerotic plaques formation by provoking autophagy in apolipoprotein E-deficient mice [167,173].

As vascular endothelial dysfunction is a premature symptom of atherosclerosis, intervention in early stages can restore the normal physiology. Matsumoto et al. evaluated the outcomes of putrescine, a precursor of spermidine and spermine, produced by the gut microbiome after administration of *Bifidobacterium animalis* spp. *Lactis LKM512* (Bifal) and *arginine* (Arg), on endothelial dysfunction [167,174]. The results indicate that the Bifal and Arg group had a lower atherosclerotic risk compared to the placebo group, by upregulating blood spermidine concentrations, which in turn promote autophagy, with improved vascular endothelial function [167].

Studies on vitamin K show promising results. Kawashima and colleagues evaluated the effects of vitamin K2, menatetrenone, on atherosclerosis and coagulation in hypercholesterolemic rabbits. Vitamin K, indispensable for the gamma-carboxylation of glutamic acid (Gla) within proteins in the body, such as vitamin K-dependent clotting factors and bone Gla-protein [175,176], in doses from 1 to 10 mg/kg/day diminished the evolution of atherosclerotic plaques, intima thickening, and pulmonary atherosclerosis, and prevented the coagulation tendency through lowering total cholesterol levels, lipid peroxidation, and factor X plasmatic activity [177].

In hypercholesterolemic rabbits, the plasmatic activity of vitamin K-dependent clotting factors is increased compared to normolipidemic animals [178,179], along with elevated procoagulant activity [177,180]. Pharmacological doses of vitamin K2 reduced the ester-cholesterol deposition at aortic sites in hypercholesterolemic rabbits [177].

Vitamin K2 supplementation for 270 days in patients with stages 3–5 of CKD significantly modified the levels of regulators of calcification, such as desphosphorylated–uncarboxylated matrix Gla protein, osteocalcin, and osteoprotegerin, diminishing the progression of atherosclerosis, but did not significantly influence the progression of calcification [181].

Osteocalcin, matrix Gla protein, and Gla-rich protein are inhibitors of calcifications of the soft tissue and require vitamin K-dependent carboxylation for activation. Circulating desphosphorylated–uncarboxylated matrix Gla protein was shown to be predictive for cardiovascular risk and mortality and the prevalence of arterial calcifications was associated with circulating total uncarboxylated matrix Gla. Over a 3-year period of supplementation of vitamin K, vascular elasticity was observed, compared to a 12% loss in the placebo group [182].

A Gla protein found in atherosclerotic plaques possesses rather controversial roles; it is linked to atherosclerotic calcification on one hand, according to Levy et al. [177,183,184], and on the other hand it can limit calcium deposits in vitro, according to Gijsbers et al. [177,185].

It is known that the gut metabolite from trimethylamine (TMA), trimethylamine-N-oxide (TMAO), is an independent risk factor for atherosclerosis. A hypothesis regarding the potential capacity of berberine to modify gut microbiota and treat diabetes, obesity, and atherosclerosis was raised, based on the inhibition of TMA/TMAO production by gut microbiota mediated by berberine. According to the work of Li Xingxing et al., berberine proved to attenuate TMA/TMAO production, so it diminished atherosclerotic lesions in animal subjects fed with cereal-based diets, supplemented with choline [186].

There is an indisputable role of diet in balancing the composition and activity of the gut microbiota. The gut microbiota and its complex composition display numerous roles, including immunomodulation. Studies on *Akkermansia muciniphila*, a mucin-degrading bacterium, were conducted due to its numerous beneficial properties on human health. Berberine intake was indicated to elevate intestinal *Akkermansia* levels and reduce atherosclerosis in ApoE -/- mice on high-fat diets. Mice fed with berberine had diminished high-fat-diet-induced atherosclerotic lesions and elevated concentrations of *Akkermansia* spp. Berberine proved to mitigate the expression of proinflammatory cytokines, reduce endotoxemia and inflammation promoted by high fat diets, and improve the gut barrier function by promoting expression of tight junction proteins. Thus, berberine induced gut changes, accentuating *Akkermansia* spp. abundance, highlighting the antiatherosclerotic effects of berberine. Similar effects were shown for resveratrol, which, by changing the gut microbiota composition, decreases TMAO-mediated atherosclerosis in ApoE -/- mice [187]. Resveratrol can restrict TMAO-mediated atherosclerosis through lowering TMAO levels and elevating bile acid secretion [187,188].

One class of microbial metabolites, known as SCFAs, represented mainly by acetate, propionate, and butyrate, which are most abundant in the human gut, were shown to function like an extra energy source, leading to de novo lipogenesis [189]. The action of SCFAs is possible after their binding to G-protein-coupled receptors, present on endothelial cells and on SMCs (in tunica media), consisting mainly of regulation of blood pressure and of the vascular tone [190]. The general effects of SCFAs might be attributed, in relation to the atherosclerotic process, to their anti-inflammatory properties by reducing migration and proliferation of immune cells, decreasing the levels of several cytokines (IL-1, IL-6 and TNF-*α*), and triggering apoptosis [191].

Another class of microbial metabolites is represented by secondary bile acids derived from primary bile acids [192]. Different in vivo and in vitro studies were carried out to draw attention to their involvement in atherosclerosis (for detailed review see [193]). Secondary biliary acids modulate bile salt hydrolase activity, which hydrolyzes glycine and taurine conjugates to liberate free biliary acids. If high quantities are present, it leads to increased cholesterol levels and foam cell formation, and it directly influences the size of atherosclerotic plaque [194]. The phenomenon is possible due to the presence of Gram-positive bacteria, such as *Bifidobacterium*, *Lactobacillus*, *Clostridium*, and *Enterococcus*, as well as Gram-negative bacteria, such as *Bacteroides* [195]. Plant-based protein intake, compared to animal-based protein intake, has been associated with decreases in *Bacteroides* and increases in *Bifidobacterium* and *Lactobacillus* concentrations, resulting in health advantages [196]. LDL cholesterol concentrations are decreased in populations consuming whole-grain foods, where *Bifidobacterium* and *Lactobacillus* are well represented and contribute to biliary acid deconjugation [197].

Although discussing all functional foods as such is considered beyond the scope of this review, we considered it more useful to synthesize recent studies, either on humans or in laboratory conditions, that establish connections between dietary components and their effects on vascular calcification, due to changes produced in the microbiome and in the microbial metabolites. The results are summarized in Table 3.

## 8. Conclusions

The very important advances in gut microbiome research recently showed its involvement in different metabolic diseases that are usually followed by the formation of atherosclerotic plaques. Bacterial metabolites, SCFA, TMAO, and biliary acids are directly affected by dietary patterns responsible for different enterotypes. Microbiome involvement in atherosclerotic plaque formation and stability should not be overlooked, since future vascular events are correlated with plaque vulnerability, undoubtedly related to its calcification. Certainly, the role of the microbiome in the prevention or management of atherosclerosis and vascular calcifications is not to be neglected. Numerous studies attest to the favorable or unfavorable effect of diet on the phenomenon of atherosclerosis. However, it is becoming clear that the microbiome is very sensitive to the composition of diets and that the products of bacterial metabolism play an overwhelming role in general health and prevention of CVDs. From now on, numerous detailed studies on the link between the microbiota and functional foods are needed. There is still much to learn regarding the connections between gut microbiome, vascular health, and cardiovascular risk, but novel therapies, including here the manipulation of diet, the use of pre/probiotics, or administration of different supplements, should be taken into consideration. Therefore, one can consider that the connections between microbiome, diet, functional foods, and the administration of pre/probiotics will represent the future means for the prevention and treatment of CVDs in an integrated context.

## Figures and Tables

**Table 1:** Influence on vascular calcifications in patients with chronic kidney disease and gut dysbiosis.

Promoters of Vascular Calcifications	Inhibitors of Vascular Calcifications
TMAO [18]	Vitamin K [19]
Inflammatory cytokines [20]	Osteoprotegerin [21]
Oxidative stress [22]	Pyrophosphate [23]
Free p-cresylsulphate [24]	Fetuin-A [21]
Serum calcium [22,23]	Matrix Gla protein [21]
Serum phosphate [22,23]	Osteopontin [23]
Serum PTH [22]	Bone morphogenetic protein 7 [21]
Serum FGF-23 [22]	
Bone morphogenetic protein 2 [21]	
Osteocalcin [21]	
Osteonectin [21] Indoxyl sulfate [25]	

**Table 2:** Dietary recommendations and their contribution to vascular calcification.

May Protect against Cardiovascular Calcifications	Potential Triggers of Cardiovascular Calcifications
Avoidance of trans fats and simple sugars [68]	Diets low in carbohydrates from young ages [69]
Diet rich in vegetables, oily fish, and long-chain omega 3 fats [68]	Plasmatic homocysteine > 12 µmol/L [70]
Calcium (800 μg/day) [68,71,72,73,74]	Oxidative stress [68]
Magnesium (≥380 mg/day) [75,76,77,78] Menaquinone (vitamin K2) [79,80]	Low serum magnesium [81]
Phylloquinone (vitamin K1) [16,82,83]	Phosphorus [84,85,86]
Serum 25(OH)D ≥ 75 nmol/L [68]	α-tocopherol [87]
High dietary potassium [88,89]	Low dietary potassium [88]
Quercetin [90] Resveratrol [91]	
Epigallocatechin gallate [92]	
Plasmatic folate > 39.4 nmol/L [68,93]	

**Table 3:** Summary of recent studies linking gut microbiome, dietary interventions, and atherosclerosis.

Study	Study Details		Treatment/Intervention		Results		
	Study Design	Subject Number	Duration	Treatment	Gut Microbiome	Microbial Metabolites	Markers of Atherosclerosis
Esgalhado, M., 2018 [162]	Randomized, double-blind, placebo-controlled trial	43 CKD patients	4 weeks	Resistant starch (Hi-Maize^®^ 260, Ingredion, Westchester, IL, USA) or placebo (manioc flour, Yoki) supplementation	↑*Bifidobacteria* with balancing the *Bacteroidetes* and *Firmicutes* ratio	↓indoxyl sulfate; p-Cresyl sulfate not affected	↓PC; ↓TBARS; ↓IL-6; ↓hs-CRP
Matsumoto, M., 2019 [174]	Randomized, double-blinded, placebo-controlled, parallel-group comparative study	44 healthy subjects	12 weeks	Normal yogurt containing Bifal and Arg or placebo (normal yogurt)	↑*Citrobacter*; ↑*Escherichia/Shigella* ratio; ↑*Enterococcus;* ↓*Bacteroidetes/Firmicutes* ratio	↑putrescine production	↑RHI; ↓BP; ↓Serum platelet; ↓triglyceride concentrations; ↑HDL-cholesterol
Li, X., 2021 [186]	Laboratory study	5 C57BL/6J strain mice and 5 ApoE KO model mice	6 and 16 weeks respectively	Standard chow diet (0.1% choline) or choline diet (chow diet with 1% additional choline) +/− BBR	the choline I group: *↑Clostridium*, *Eubacterium*, *Lachnoclostridium*, *Roseburia*, *Odoribacter;* the C + BBR group: ↑*Bacteroides*, *Prevotella*, *Parabacteroides*, *Alloprevotella*	↓TMA	↓serum TMAO level; ↓progression of atherosclerotic plaque; ↓macrophage-specific biomarkers of macrophage-derived foam cells
Chen, M., 2016 [188]	Laboratory study	10 female C57BL/6J strain mice and 10 ApoE model mice	30 days	Standard chow diet (NIH31 modified mouse/rat diet) or resveratrol	↑*Lactobacillus*; ↑*Bifidobacterium*; ↑*Bacteroides*; ↑*Akkermansia*	↓TMA ↑BA deconjugation	↓serum TMAO level; ↑hepatic BA neosynthesis; ↓TMAO-induced atherosclerosis

CKD—chronic kidney disease, PC—protein carbonyl, TBARS—thiobarbituric-acid-reactive substances, IL-6—interleukin-6, hs-CRP—high-sensitivity C-reactive protein, RHI—reactive hyperemia index, BP—blood pressure, BBR—berberine, TMA—trimethylamine, TMAO—trimethylamine N-oxide, BA—bile acid, ↑—elevation, ↓—diminution.
